# Comparison of 21-Plex PCR and API 20C AUX, MALDI-TOF MS, and rDNA Sequencing for a Wide Range of Clinically Isolated Yeast Species: Improved Identification by Combining 21-Plex PCR and API 20C AUX as an Alternative Strategy for Developing Countries

**DOI:** 10.3389/fcimb.2019.00021

**Published:** 2019-02-15

**Authors:** Amir Arastehfar, Farnaz Daneshnia, Mohammad Kord, Maryam Roudbary, Hossein Zarrinfar, Wenjie Fang, Sayed Jamal Hashemi, Mohammad Javad Najafzadeh, Sadegh Khodavaisy, Weihua Pan, Wanqing Liao, Hamid Badali, Sassan Rezaie, Kamiar Zomorodian, Ferry Hagen, Teun Boekhout

**Affiliations:** ^1^Yeast Biodiversity Department, Westerdijk Fungal Biodiversity Institute, Utrecht, Netherlands; ^2^Department of Medical Parasitology and Mycology, Tehran University of Medical Sciences, Tehran, Iran; ^3^Department of Medical Mycology and Parasitology, School of Medicine, Iran University of Medical Sciences, Tehran, Iran; ^4^Allergy Research Center, Mashhad University of Medical Sciences, Mashhad, Iran; ^5^Shanghai Key Laboratory of Molecular Medical Mycology, Department of Dermatology, Shanghai Institute of Medical Mycology, Shanghai Changzheng Hospital, Second Military Medical University, Shanghai, China; ^6^Food Microbiology Research Center, Tehran University of Medical Sciences, Tehran, Iran; ^7^Department of Parasitology and Mycology, School of Medicine, Mashhad University of Medical Sciences, Mashhad, Iran; ^8^Zoonoses Research Center, Kurdistan University of Medical Sciences, Sanandaj, Iran; ^9^Department of Medical Mycology, Invasive Fungi Research Center, School of Medicine, Mazandaran University of Medical Sciences, Sari, Iran; ^10^Department of Medical Mycology and Parasitology, Basic Sciences in Infectious Diseases Research Center, School of Medicine, Shiraz University of Medical Sciences, Shiraz, Iran; ^11^Yeast Biodiversity Department, Institute of Biodiversity and Ecosystem Dynamics, University of Amsterdam, Amsterdam, Netherlands

**Keywords:** API 20C AUX, 21-plex PCR, MALDI-TOF MS, LSU rDNA sequencing, developing countries

## Abstract

Occurrence of non-*Candida albicans Candida* (NCAC) species that are associated with elevated MIC values and therapeutic failures are increasing. As a result, timely and accurate means of identification to the species level is becoming an essential part of diagnostic practices in clinical settings. In this study, 301 clinically isolated yeast strains recovered from various anatomical sites [Blood (*n* = 145), other sites (*n* = 156)] were used to assess the accuracy and practicality of API 20C AUX and 21-plex PCR compared to MALDI-TOF MS and large subunit rDNA (LSU rDNA). MALDI-TOF MS correctly identified 98.33% of yeast isolates, 100% of top five *Candida* species, 95.7% of rare yeast species, while 1.3% of isolates were misidentified. API 20C AUX correctly identified 83.7% of yeast isolates, 97.2% of top five *Candida* species, 61.8% of rare yeast species, while 16.2% of yeast isolates were misidentified. The 21-plex PCR, accurately identified 87.3% of yeast isolates, 100% of top five *Candida* species, 72% of rare yeast species, but it misidentified 1.3% of rare yeast species while 9.9% of whole yeast isolates were not identified. The combination of rapidity of 21-plex PCR and comprehensiveness of API 20C AUX, led to correct identification of 92% of included yeast isolates. Due to expensiveness of MALDI-TOF MS and sequencing, this combination strategy could be the most accurate and inexpensive alternative identification strategy for developing countries. Moreover, by the advent and development of cost-effective, reliable, and rapid PCR machines that cost 130 US dollars, 21-plex could be integrated in routine laboratories of developing and resource-limited countries to specifically identify 95% causative agents of yeast-related infections in human. Databases of MALDI-TOF MS, API 20C AUX, and the number of target species identified by 21-plex require further improvement to keep up with the diverse spectrum of yeast species.

## Introduction

Increasing population of immunocompromised patients and administration of broad-spectrum antibiotics etc. (Pappas, 2006), led to a higher occurrence of fungal infections in clinical settings (Yapar, 2014). Among opportunistic yeast species, *Candida albicans* is continuously reported to be the most commonly encountered yeast species (Pappas et al., 2010). However, applying changes to the clinical practices and interventions resulted in epidemiological landscape and emergence of non-*Candida albicans Candida* (NCAC) species (Pham et al., 2014). For instance, since the introduction of echinocandins as a prophylactic antifungal, selective pressure has aided in emergence of NCAC species that are less susceptible to this class of antifungals (Pham et al., 2014). Moreover, more frequent isolation of yeast species exhibited inherent less susceptibility/acquired resistance to fluconazole and those with multi-drug resistant traits (MDR) highlight the importance of correct identification (Pfaller et al., 2008; Bizerra et al., 2014; Pham et al., 2014; Chowdhary et al., 2016). Due to the availability of trifle classes of antifungals, monitoring frequency, and epidemiology of yeast species would become an imperative practice in clinical routine laboratories.

Traditionally, phenotypic assays such as direct microscopy, biochemical characterization, and culture are among the most widely used technique to identify yeast species (Posteraro et al., 2015). API 20C AUX, Vitek2 YST ID Card, and AuxaColor are among the most widely exploited biochemical means of identification (Posteraro et al., 2015; Zhao et al., 2017). However, these techniques are time-consuming, labor-intensive, and expensive (Posteraro et al., 2013). Many studies showed that API kits cannot reliably identify rare yeast species, which are less susceptible to routinely used antifungals (Castanheira et al., 2013; Magobo et al., 2014). On the contrary, Sanger sequencing of common barcoding regions and MALDI-TOF MS proved to be the most accurate identification tools (Criseo et al., 2015). Although, these techniques are used in routine laboratories in developed countries, they are regarded as unaffordable devices in developed countries (Posteraro et al., 2013; Criseo et al., 2015; Aslani et al., 2018).

Advances in the machinery of polymerase chain reaction (PCR) has made this device as an affordable identification tool for developing and low-resourced countries. Moreover, due to showing a reasonable reproducibility, WHO recommended PCR as a reliable identification tool in developing countries (Ragheb and Jimenez, 2014). Although, commercial PCR machines still are considered to be expensive (2000–4000 US dollars), it has been shown that using the most basic off-the-shelf tools, it is possible to develop reliable, rapid, and cheap (130 US dollars) PCR machines that without continuous power supply and as efficient as commercial PCR machines can amplify PCR products larger than 1,509 bps (Wong et al., 2015). These kinds of PCR machines can be easily made and expanded as an in on-site diagnostic tools in resource-limited countries (Wong et al., 2015). Unfortunately, there are few PCR-based techniques that can target a comprehensive list of opportunistic yeast species. Automated rep-PCR proved to be a reliable, but expensive assay (Zhao et al., 2017). Recently, Arastehfar et al., have developed a 21-plex PCR assay that targets the most clinically important yeast species, which uses the basic chemistry and devices that are used in routine laboratories (Arastehfar et al., 2018). As 21-plex intended to be used in developing countries, we evaluated its practicality and accuracy compared to a time-consuming and widely used biochemical technique in developing countries, namely API 20C AUX, MALDI-TOF MS, and sequencing of large subunit D1/D2 domains of rDNA (LSU rDNA) as a gold standard.

## Materials and Methods

### Ethical Approval

Due to the diagnostic nature of this study, and the fact that we did not include any clinical samples (blood, serum, CSF, urine, etc…) or biopsies derived from patients, we did not have any consent forms from patients. Isolates investigated in this study were part of previous studies that had been approved by the local ethical committees of Mashhad University of Medical Sciences and Tehran University of Medical Sciences under the following ethical code numbers IR MUMS fm REC.1397.268, and IR. TUMS. .SPH.REC.1396.4195. As such, inclusion of clinical strains in our study did not require institutional ethical approval according to institutional and national guidelines.

### Isolates and Growth Conditions

Two hundred and ninety-eight clinical yeast strains encompassing a wide range of yeast species that were recovered from clinical sources [blood (*n* = 145), vagina (*n* = 71), sputum (*n* = 35), oral swabs (*n* = 21), Cerebrospinal fluid (CSF) (*n* = 8), urine (*n* = 7), nail (*n* = 6), tube aspirate (*n* = 2), penis (*n* = 1), and throat (*n* = 1)] were retrospectively collected from Iran and China (Table 1). Due to the importance of *Candida auris* as an emerging yeast species, and lack of this species in our clinical collection, three CBS reference strains were included. These 301 strains were serially numbered from 1 to 301, prepared as a blinded test set, and three centers were involved for their identification. In the Netherlands two technicians separately performed MALDI-TOF MS and API 20C AUX, In China sequencing of D1/D2 domains of rDNA was carried out, and in Iran, as an example of a developing country a multiplex PCR known as 21-plex was utilized. Strains comprised a diverse range of yeast species, including most and less prevalent *Candida* species and basidiomyceteous yeasts, including *Trichosporon, Cryptococcus*, and *Rhodotorula*. Strains were grown on GYPA and SDA media for 48 h at 25°C, single colonies were struck on SDA and GYPA media, incubated another 48 h at 25°C, and from those pure cultures identifications were performed.

**Table 1:** Summary of species utilized in this along with their source of isolation and the country of origin.

**Species (number)**	**Source of isolation**	**Country of origin**
*Candida albicans* (*n =* 59)	Blood (*n* = 52), Vagina (*n* = 6), Oropharyngeal (*n* = 1)	Iran
*Candida glabrata* (*n* = 43)	Vagina (*n* = 20), Blood (*n* = 18), Urine (*n* = 2), sputum (*n* = 1), Skin (*n* = 1), Penis (*n* = 1)	Iran
*Candida parapsilosis* (*n* = 40)	Blood (*n* = 36), Vagina (*n* = 3), Nail (*n =* 1)	Iran
*Candida tropicalis* (*n* = 14)	Blood (*n* = 13), Nail (*n =* 1)	Iran
*Pichia kudriavzevii (Candida krusei)* (*n* = 27)	Vagina (*n* = 20), Blood (*n* = 5), Throat (*n =* 1), Urine (*n* = 1),	Iran
*Candida dubliniensis* (*n* = 3)	Blood (*n* = 3)	Iran
*Kluyveromyces marxianus* (*Candida kefyr*) (*n* = 27)	Oropharyngeal (*n* = 13), Sputum (*n =* 6), Vagina (*n* = *5*), Urine (*n* = *2*), Blood (*n* = 1)	Iran
*Clavispora lusitaniae* (*Candida lusitaniae*) (*n* = 21)	sputum (*n* = 7), Oropharyngeal (*n* = 6), Blood (*n* = 5), Tracheal Tube (*n* = 2), Nail (*n =* 1),	Iran, China
*Meyerozyma guilliermondii* (*Candida guilliermondii*) (*n* = 14)	Blood (*n* = 5), Sputum (*n =* 5), Nail (*n =* 1), Oropharyngeal (*n* = 1), Urine (*n* = 1), Vagina (*n* = 1)	Iran, China
*Kodamaea ohmerii* (*n* = 10)	Sputum (*n =* 10)	China
*Candida metapsilosis* (*n* = 2)	Sputum (*n =* 2)	China
*Candida orthopsilosis* (*n* = 4)	Blood (*n* = 2), Sputum (*n =* 1), Nail (*n =* 1)	Iran
*Candida bracarensis* (*n* = 1)	Sputum (*n =* 1)	China
*Cyberlindnera fabianii* (*Candida fabianii*) (*n* = 5)	Vagina (*n* = 4), Blood (*n* = 1)	Iran
*Cyberlindnera jadinii* (*Candida utilis*) (*n* = 1)	Vagina (*n* = 1)	Iran
*Candida auris* (*n* = 3)	Reference strains (*n* = 3)	Westerdijk Collection (CBS12883, CBS12884, and CBS12885)
*Candida inconspicua* (*n* = 1)	Vagina (*n* = 1)	Iran
*Pichia cactophila* (*n* = 4)	Vagina (*n* = 4)	Iran
*Candida vulnera* (*n* = 1)	Vagina (*n* = 1)	Iran
*Kluyveromyces lactis* (*n* = 1)	Vagina (*n* = 1)	Iran
*Diutina pararugosa* (*Candida pararugosa*) (*n* = 1)	Vagina (*n* = 1)	Iran
*Saccharomyces cerevisiae* (*n* = 1)	Nail (*n =* 1)	Iran
*Rhodotorula mucilaginosa* (*n* = 2)	Vagina (*n* = 2)	Iran, China
*Cryptococcus neoformans* (*n* = 6)	CSF (*n* = 5), Blood (*n* = 1)	Iran,
*Cryptococcus gattii* (*n* = 3)	CSF (*n* = 3)	China
*Cryptococcus saitoi* (*n* = 1)	Blood (*n* = 1)	Iran
*Trichosporon asahii* (*n* = 1)	Urine (*n* = 1)	Iran
*Trichosporon asteroides* (*n* = 2)	Blood (*n* = 2)	Iran, Fang
*Trichosporon mucoides* (*n =* 1)	Sputum (*n =* 1)	China
*Trichosporon faecale* (*n =* 1)	Sputum (*n =* 1)	China
*Cutaneotrichosporon curvatus* (*n* = 1)	Vagina (*n* = 1)	Iran
Total (*n* = 301)		

*Except for Candida auris that was ordered from the collection of Westerdijk Institute, the rest of species were collected from patient materials*.

### DNA Extraction

One full loop of pure colonies (with the volume of 10 μl) was suspended in 100 μl of TaKaRa Lysis buffer (TaKaRa, Japan), vortexed thoroughly, and incubated at 95°C for 30. After 15 min incubation at 95°C, lysates were vortexed vigorously and incubation for another 15 min at 95°C was continued. In the last step, lysates were vortexed again and centrifuged at 14,000 rpm for 5 min. Two microliters of obtained supernatants were used as the PCR template.

### Sequencing

One technician was responsible for performing sequencing of D1/D2 domains of rDNA (LSU rDNA), as described previously (Stielow et al., 2015). Bidirectional chain Terminated Sanger sequencing using referenced primers were performed. Obtained sequences were searched in BLAST database (https://blast.ncbi.nlm.nih.gov) and the identity of each strain was assigned accordingly. This experiment was carried out in China.

### Stepwise 21-Multiplex PCR

Identification of 301 using 21-plex PCR was performed in Iran. This technique contains three multiplex PCR reactions, with the first one identifying the most prevalent *Candida* species (Table 2), the second one targeting rare *Candida* species, and the third multiplex reaction identifying the most clinically important basidiomyceteous yeast species, i.e., *Trichosporon, Cryptococcus, Geotrichum*, and *Rhodotorula* (Arastehfar et al., 2018). Authors claimed that except for *Candida zeylanoides*, the rest of target species were correctly identified. PCR reaction and program were used same same as described, previously (Arastehfar et al., 2018). PCR products were run on 2% agarose gel (voltage of 135, 60 min), stained with Gel Red (BioTium Corporation, USA), and visualized by Gel Doc (Gel Doc XR^+^, BioRad, California, USA). This experiment was performed in Iran.

**Table 2:** Summary of species identification of wide range of clinically obtained yeast species using three approaches, MALDI-TOF MS, 21-plex, API 20C AUX, and their comparison with large subunit of rDNA domain sequencing.

	**Correctly identified**			**Misidentified**			**Not-identified**		
**Species (number)**	**MALDI-TOF** **MS**	**21-plex**	**API 20C AUX**	**MALDI-TOF** **MS**	**21-plex**	**API 20C AUX**	**MALDI-TOF** **MS**	**21-plex**	**API 20C AUX**
*Candida albicans* (*n =* 59)	59	59	59	–	–	–	–	–	–
*Candida glabrata* (*n =* 42)	43	43	43	–	–	–	–	–	–
*Candida parapsilosis* (*n =* 40)	40	40	40	–	–	–	–	–	–
*Candida tropicalis* (*n =* 14)	14	14	14	–	–	–	–	–	–
*P. kudriavzevii* (*n =* 27)	27	27	22	–	–	–	–	–	–
*Candida dubliniensis* (*n =* 3)	3	3	2	–	–	–	–	–	–
*Kluyveromyces marxianus* (*n =* 27)	27	27	24	–	–	–	–	–	–
*Clavispora lusitaniae* (*n =* 21)	19	21	17	2	–	4	–	–	–
*Meyerozyma guilliermondii* (*n =* 14)	12	14	11	2	–	3	–	–	–
*Kodamaea ohmerii* (*n =* 10)	10	–	9	–	–	1	–	10	–
*Candida metapsilosis* (*n =* 2)	2	–	–	–	–	2	–	2	–
*Candida orthopsilosis* (*n =* 4)	4	–	–	–	4	4	–	–	–
*Candida bracarensis* (*n =* 1)	1	–	–	–	–	1	–	1	–
*Cyberlindnera fabianii* (*n =* 5)	5	–	–	–	–	5	–	5	–
*Cyberlindnera jadinii* (*n =* 1)	1	–	1	–	–	–	–	1	–
*Candida auris* (*n =* 3)	3	3	–	–	–	3	–	–	–
*Candida inconspicua* (*n =* 1)	1	–	–	–	–	1	–	1	–
*Pichia cactophila* (*n =* 4)	4	–	–	–	–	4	–	4	–
*Candida vulnera* (*n =* 1)	1	–	–	–	–	1	–	1	–
*Kluyveromyces lactis* (*n =* 1)	1	–	–	–	–	1	–	1	–
*Diutina pararugosa* (*n =* 1)	1	–	–	–	–	1	–	1	–
*Saccharomyces cerevisiae* (*n =* 1)	1	–	1	–	–	–	–	1	–
*Rhodotorula mucilaginosa* (*n =* 2)	2	2	2	–	–	–	–	–	–
*Cryptococcus neoformans* (*n =* 6)	6	6	6	–	–	–	–	–	–
*Cryptococcus gattii* (*n =* 3)	3	3	–	–	–	3	–	–	–
*Cryptococcus saitoi* (*n =* 1)	1	–	–	–	–	1	–	1	–
*Trichosporon asahii* (*n =* 1)	1	1	1	–	–	–	–	–	–
*Trichosporon asteroides* (*n =* 2)	2	2	–	–	–	2	–	–	–
*Trichosporon mucoides*	1	–	–	–	–	1	–	1	–
*Trichosporon faecale*	1	1	–	–	–	1	–	–	–
*Cutaneotrichosporon curvatus* (*n =* 1)	–	1	–	–	–	1	1	-	–
Total (*n =* 300)	296 (98.33%)	267 (88.7%)	251 (83.7%)	4 (1.32%)	4 (1.3%)	49 (16.27%)	0.33%	30 (9.96%)	0

### MALDI-TOF MS

Full-extraction method as it was utilized as suggested, previously (Marklein et al., 2009), and identification was carried out by using Microflex LT, MALDI-TOF MS device (Bruker Daltonics, Bremen Germany). Scores lower than 1.7, ≥1.7 <2, and above 2 were considered as not reliable identification, identification at the genus level, and identification at the species level, respectively. MALDI-TOF MS was performed in Netherlands.

### API 20C AUX

API 20C AUX (BioMerieux, France), based on assimilation of 19 sugars and presence or absence of hyphal/pseudohyphal formation identifies clinically important yeast species. API strips were prepared as suggested by the manufacturer and incubated at 30°C for 48–72 h. Besides of results obtained from sugar assimilation profile, the possibility of hyphal/pseudohyphal formation was investigated as described previously (Keçeli et al., 2016). As 72 h incubation of API strips improved the accuracy of results (Willemsen et al., 1997), final sugar assimilation patterns were read after 72 h incubation at 25°C. Accurate identification was based on identity and T indices greater than 90% and 0.75, respectively. For hints lower than those values, the first proposed identity was assigned as the species name. API 20C AUX experiments were carried out in the Netherlands.

### Statistical Analysis

The strength of agreements between API 20C AUX and sequencing, 21-plex PCR and sequencing, MALDI-TOF MS and sequencing was assessed by Kappa coefficient value. Kappa coefficient value was calculated by SPSS v.23 software (Chicago, USA).

## Results

### Comparative Analysis of LSU rDNA Sequencing and MALDI-TOF MS

Two hundred and ninety-six of isolates (98.33%) were correctly identified, four isolates (1.3%) misidentified, and only one isolate was not identified using MALDI-TOF MS (Table 2). All of those yeast isolates were identified with the score of over two, indicating reliable identification at the species level. Surprisingly, two isolates of *Meyerozyma guilliermondii* and two isolates of *Clavispora lusitaniae* were misidentified as *C. dendronema* and *Wickerhamiela pagnoccae* (Table 3), respectively. Despite of repeated efforts and experiments using full-extraction method, the same results were obtained. All of top 5 *Candida* species [*C. albicans, C. glabrata, C. parapsilosis, C. tropicalis*, and *Pichia kudriavzevii* (*Candida krusei*)] and 95.7% of rare yeast species were identified, correctly. In total, MALDI-TOF MS identified 98.33% of yeast isolates. The Kappa coefficient value for MALDI-TOF MS and sequencing was 0.991.

**Table 3:** Misidentified isolates using MALDI-TOF MS, 21-plex, and API 20C AUX compared to LSU rDNA sequencing as the gold standard method.

**Species**	**Misidentified as**		
	**MALDI-TOF MS**	**21-plex**	**API 20C AUX**
*Pichia kudriavzevii* (*n =* 27)	–	–	*Candida norvegensis* (*n =* 4), *Cryptococcus humicola* (*n =* 1)
*Candida dubliniensis* (*n =* 3)	–	–	*Candida albicans* (*n =* 1)
*Kluyveromyces marxianus* (*n =* 27)	–	–	*Rhodotorula mucilaginosa* (*n =* 2) and *Candida albicans* (*n =* 1)
*Clavispora lusitaniae* (*n =* 21)	*Wickerhamiela pagnoccae* (*n =* 2)	–	*Candida tropicalis*(*n =* 2), *Candida parapsilosis* (*n =* 1) and *Candida albicans* (*n =* 1)
*Meyerozyma guilliermondii* (*n =* 14)	*Candida dendronema* (*n =* 2)	–	*Candida lusitaniae* (*n =* 3)
*Kodamaea ohmerii* (*n =* 10)	–	–	*Candida guilliermondii* (*n =* 1)
*Candida metapsilosis* (*n =* 2)	–	–	*Candida parapsilosis* (*n =* 2)
*Candida orthopsilosis* (*n =* 4)	–	*C. parapsilosis* (*n =* 4)	*Candida parapsilosis* (*n =* 4)
*Candida bracarensis* (*n =* 1)	–	–	*Candida glabrata* (*n =* 1)
*Cyberlindnera fabianii* (*n =* 5)	–	–	*Candida utilis* (*n =* 3), *Candida pelliculosa* (*n =* 1), *Candida thermophila* (*n = 1*)
*Candida auris* (*n =* 3)	–	–	*Rhodtorula glutinis* (*n =* 3)
*Candida inconspicua* (*n =* 1)	–	–	*Trichosporon asahii* (*n =* 1)
*Pichia cactophila* (*n =* 4)	–	–	*Candida norvegensis* (*n =* 2), *Candida ciferrii* (*n =* 1), *Candida spherica* (*n =* 1)
*Candida vulnera* (*n =* 1)	–	–	*Kodamaea ohmeri* (*n =* 1)
*Kluyveromyces lactis* (*n =* 1)	–	–	*Candida kefyr* (*n =* 1)
*Candida pararugosa* (*n =* 1)	–	–	*Candida rugosa* (*n =* 1)
*Cryptococcus gattii* (*n =* 3)	–	–	*Cryptococcus neoformans* (*n =* 3)
*Cryptococcus saitoi* (*n =* 1)	–	–	*Cryptococcus albidus* (*n =* 1)
*Trichosporon asteroides* (*n =* 2)	–	*Trichosporon asahii* (*n =* 2)	*Trichosporon asahii* (*n =* 2)
*Trichosporon mucoides* (*n =* 1)	–	–	*Trichosporon asahii* (*n =* 1)
*Trichosporon faecale* (*n =* 1)	–	*Trichosporon asahii* (*n =* 1)	*Trichosporon asahii* (*n =* 1)
*Cutaneotrichosporon curvatus* (*n =* 1)	–	*Trichosporon asahii* (*n =* 1)	*Trichosporon asahii* (*n =* 1)
Total number of misidentified isolates	*n =* 4	*n =* 8	*n =* 49

### Comparative Analysis of LSU rDNA Sequencing and 21-Plex PCR

Two hundred and sixty-seven isolates (88.7%) were correctly identified, 4 isolates (1.3%) were misidentified, and 29 isolates (9.96%) were not identified by the 21-plex PCR technique (Table 2). Both misidentified and none-identified isolates were rare yeast species (Tables 2, 3), while all of top 5 *Candida* species (*C. albicans, C. glabrata, C. parapsilosis, C. tropicalis*, and *P. kudriavzevii*) were identified, correctly. The Kappa coefficient value for 21-plex PCR and sequencing was 0.943.

### Comparative Analysis of LSU rDNA Sequencing and AP 20C AUX

Two hundred and fifty-one isolates (83.7%) were correctly identified, 49 (16.2%) isolates were misidentified, and there was no species without identification using API 20C AUX (Table 2). The majority of misidentified yeast isolates were among rare species (*n* = 45), and only 4 strains of *P. kudriavzevii* were among top 5 *Candida* species (Table 3). API 20C AUX showed the lowest Kappa Coefficient value (0.918) when compared to sequencing.

### Combined Strategy (API 20C AUX and 21-Plex PCR) for Identification of Yeast Collection

Although, the 21-plex PCR showed higher accuracy, API 20C AUX more comprehensively identified yeast species. For instance, species such as *Saccharomyces cerevisiae* (1/1) and *Kodamaea ohmerii* (9/10) were correctly identified by API 20C AUX, while they were not identified by 21-plex PCR. By integrating the rapidity of PCR and comprehensiveness of API 20C AUX we could correctly identify 92% of yeast isolates included in our study. As 21-plex PCR represented a fast and reliable technique for the majority of the more prevalent yeast species and API 20C AUX requires 48–72 h for identification, we used 21-plex as the first line and rapid identification tool and in case of encountering with negative results, API 20C AUX could be used as the alternative technique.

## Discussion

Because the number of yeast species causing infection in human is increasing, fast, and accurate identification of clinically obtained isolates is highly important to initiate appropriate antifungal regimen (Pincus et al., 2007). Sequencing of commonly used phylogenetic markers, MALDI-TOF MS, PCR-based techniques, and biochemical and phenotypic assays are considered as the most popular identification systems. Herein, we have compared the accuracy of the API 20C AUX and 21-plex PCR methods in the light of MALDI-TOF MS and sequencing of D1/D2 domains of rDNA.

In our study, MALDI-TOF MS showed a good accuracy for identification of a diverse range of opportunistic yeast species (98.3%). All of five top *Candida* species (*C. albicans, C. glabrata, C. parapsilosis, C. tropicalis*, and *P. kudriavzveii*) and 95.7% of rare yeast species were identified successfully (Kappa value of 0.991). Despite the close genetic background of cryptic species complexes, MALDI-TOF MS identified them to the species level (note that we did not have *C. africana*). This is in agreement with other studies, where cryptic species complexes of *C. albicans, C. glabrata*, and *C. parapsilosis* were correctly identified (Santos et al., 2011). Moreover, FDA-approved spectra of wide-spreading multidrug-resistant yeast species, i.e., *Candida auris*, has been added to the clinical database of MALDI-TOF MS, leading to rapid and reliable identification of this organism (Bao et al., 2018). Although, cases of misidentification for *Cryptococcus* and *Trichosporon* species had been reported previously (Kolecka et al., 2013; Sendid et al., 2013; Ling et al., 2014; Zhao et al., 2017), except for *Cutaneotrichosporon curvatus*, all of our clinical isolates of aforementioned species were correctly identified (the reference spectra of this species is not included in the MALDI-TOF MS database). Consistent with the other studies and due to hardship of obtaining proper spectra for *Meyerozyma guilliermondii* (Ling et al., 2014; Zhao et al., 2017), using MALDI-TOF MS *M. guilliemondii* (*n* = 2/14) and *Cl. lusitaniae* (*n* = 2/21) were misidentified in our study. MALDI-TOF MS, despite of being fast, robust, and providing accurate strain identity is still considered as an economical burden, especially for developing countries, not only to purchase the device but also as it requires trained technicians and periodical maintenances (Posteraro et al., 2013; Criseo et al., 2015; Aslani et al., 2018). Due to occurrence of misidentification of some rare yeast species in our study and the other studies (Sendid et al., 2013; Zhao et al., 2017), improvement of the MALDI-TOF MS library can enhance the accuracy of this technique.

Although Vitek 2 YST ID Card reported amongst the most popular biochemical assays used in routine laboratories (Posteraro et al., 2015), API 20C AUX showed a higher agreement with sequencing of ITS and D1/D2 domains of rDNA (Zhao et al., 2017). As a result, herein API 20C AUX was used as the representative of biochemical assays. In our study, API 20C AUX correctly identified 83.7% of all included yeast isolates, the vast majority (97.26%) of the most prevalent *Candida* species (*C. albicans, C. glabrata, C. parapsilosis, C. tropicalis*, and *P. kudriavzveii*) and 61.8% of rare yeast species which is consistent with previous studies (Keçeli et al., 2016; Zhao et al., 2017) (Kappa value of 0.918). Despite the fact that, API 20C AUX misidentified the majority (4/5) of *Pichia kudriavzevii* strains as *Pichia norvegensis*, studies have revealed that both species are inherently resistant to fluconazole and genetically are close to each other (Sandven et al., 1997; Musso et al., 2014). Although, identification to the species level is an integral part of epidemiological studies, grouping of few species with the same antifungal susceptibility pattern in routine settings may hasten the timely administration of antifungals and as a consequence it may contribute to a lower the mortality rate. For instance, *C. albicans, C. parapsilosis, C. tropicalis*, and *C. dubliniensis* are all susceptible to fluconazole, and hence, using one probe they were identified as the fluconazole susceptible group (McMullan et al., 2008). On the other hand, *in vitro* studies have shown that *C. dubliniensis* compared to *C. albicans* more rapidly can acquire resistance to antifungals(Moran et al., 1997), underscoring the importance of specie level identification in such circumstances.In our study, 66.6% (2/3) of *C. dubliniensis* strains were correctly identified, whereas other cryptic complex species of *C. metapsilosis, C. orthospsilosis*, and *C. bracarensis* were all identified as *C. parapsilosis* and *C. glabrata*, which is in agreement with other reports (Keçeli et al., 2016; Zhao et al., 2017). For other clinically rare *Candida* species including *M. guilliermondii, Cl. lusitaniae*, and *Kl. marxianus* we observed few misidentification cases. All CBS reference strains of *C. auris* in concordance with other studies were misidentified as *Rhodotorula glutinis* (Magobo et al., 2014). In our setting, application of API 20C AUX for the most prevalent basidiomyceteous yeasts did not obtain satisfactory results for *Cr. gattii, Trichosporon mucoides*, and *T. asteroides* as seen with the previous studies (Guo et al., 2011). Biochemical assays in general and API 20C AUX in particular, despite of generating satisfactory results for rare yeast species, are labor-intensive, time-consuming, and interpretation of sugar assimilation profiles is sometimes subjective. Moreover, in order to generate accurate identity, API 20C AUX requires further testing of yeast isolates for hyphal/pseudohyphal formation (Guo et al., 2011). Numerous of reports have shown that biochemical assays can lead to underestimation of some rare yeast species and ignoring them as etiological agents of infection in human (Kathuria et al., 2015; Svobodova et al., 2016). As an example, all biochemical assays provide inaccurate identity for *C. auris* and it is mistaken for other yeast species such as *C. parapslosis, C. famata, Rhodotorula glutinis etc* (Kordalewska et al., 2017), leading to its persistence as a colonizer in hospital environment and source of future outbreaks. Given the rise in occurrence of rare yeast species that are less susceptible to fluconazole (Miceli et al., 2011), this could be of a great importance, as in developing countries due to limited economical support, this drug is administered as the drug of choice for the first line therapy (Kordalewska et al., 2018). Consequently, for a species like *C. auris* that exhibited resistance to all classes of antifungals, especially more than 90% resistance to fluconazole (Kathuria et al., 2015), along with high rate of mortality of 30–60% (Chowdhary et al., 2017), such misidentifications could be accompanied by adverse consequences.

Recently, we have developed a multiplex PCR that in a stepwise manner identifies the majority of yeast species regularly encountered in clinical settings as the cause of infection in human (Arastehfar et al., 2018). With the application of 21-plex PCR we correctly identified 87.3% of all included yeast species, 100% of most prevalent *Candida* species and 72% of rare yeast species (Kappa value of 0.943). As this assay originally was not intended for identification of other rare yeast species, such as *S. cerevisiae, Cyberlindnera fabianii, Cyberlindnera jadinii, Kodamaea ohmerii, C. cactophila, C. norvegensis, Kl. lactis, Cryptococcus saitoi*, and *T. mucoides*, they were not identified, accordingly. This assay showed a high degree of specificity (98.7%). Although, *T. asteroides* (*n* = 2)*, T. faecale* (*n* = 1), and *Cutaneotrichosporon curvatus* (*n* = 1) were identified as *T. asahii*, 21-plex utilizes one universal primer to identify most clinically important *Trichosporon* species in the genus level, and hence, these cases were not considered as misidentification. Although, a slight difference in susceptibility pattern of triazoles (ravuconazole, itraconazole, and voriconazole) and AMB between *T.asahii* and non-*T. asahii* strains have been observed (Paphitou et al., 2002), MIC values are not always correlated with clinical outcomes (Paphitou et al., 2002). Accordingly, in order to prove the difference in clinical outcomes, *in vivo* testing with neutropenic and immunocompromised mice is still required. As a result, identification of species of non-*T. asahii* and *T. asahii* to the genus level (only as *Trichosporon*) will be clinically relevant, unless otherwise is proved. In concordance with sequencing of D1/D2 domains of rDNA and MALDI-TOF MS, all included strains of *C. auris* were correctly identified. Other PCR-based techniques such Rep-PCR shown to be a robust technique to identify a wide range of yeast species (Pincus et al., 2007; Zhao et al., 2017), but it requires tedious DNA extraction methods, capillary electrophoresis for separation of amplified PCR product, and highly trained personnel (Pincus et al., 2007; Zhao et al., 2017). Moreover, using rep-PCR technique, identification of a single isolate requires 90 USD, while in our setting using 21-plex costs 0.75–1 euros. Although 21-plex PCR exhibited a high degree of sensitivity (98.7%), there were some species (*K. ohmerii, C. metapsilosis*, and *C. bracarensis, Cy. fabianii* and *Cr. satoi*) that were not identified. As a result, including more multiplex PCR assays to identify other clinically important yeast species, can improve the sensitivity of 21-plex PCR.

Sole dependence on phenotypic assays could result in misidentification and subsequently oblivion of emerging and important yeast species, while combination of comprehensiveness of biochemical and phenotypic assays with the rapidity of PCR-based techniques (21-plex PCR) could increase the accuracy of identification, reduces required time and expenses, and circumvent the imperfection of either assays. For instance, despite of obtaining negative results using the 21-plex PCR for strains of *K. ohmerii, Cy. jadinii, S. cerevisiae*, they were correctly identified by API 20C AUX. Moreover, 21-plex PCR due to its rapidity and possessing high specificity and sensitivity, if accompanied by inexpensive and reliable PCR devices, it could be used as a reliable means of identification in developing and resource-limited countries. This could be relevant for routine laboratories, especially in developing countries, where robust and accurate means of identification such as MALDI-TOF MS and Sanger sequencing are lacking. In terms of required expenses, MALDI-TOF MS was the least expensive (less than 0.3 Euros), followed by 21-plex PCR (0.75–1 Euros/reaction), sequencing (3 Euros) and API 20C AUX (5.9 Euros/reaction). As a result, API 20C AUX was the most expensive and least accurate identification tool.

Despite the fact that we included various rare *Candida* and yeast species obtained from multiple healthcare facilities in Iran and China, our study still could benefit from addition of other rare yeast species, such *Debaromyces hansinii, Diutina rugose, Yarrowia lipolytica*, and the like. Moreover, we did not obtain clinical isolates of cryptic species complexes of *C. nivariensis* and *C. africana* and hence we could not observe how well they could be differentiated from *C. glabrata* and *C. albicans* using applied techniques.

## Author Contributions

AA, FD, SK, WP, MK, and TB have designed the study, did the experiments, and participated in draft preparation and revision. MR, HZ, WF, MN, WL, SR, HB, KZ, and FH have provided the isolates, participated in carrying out the experiments, and assisted in paper revision. SH provided strains and participated in performing 21-plex PCR.

### Conflict of Interest Statement

The authors declare that the research was conducted in the absence of any commercial or financial relationships that could be construed as a potential conflict of interest.
