# Corrigendum: Comparison of 21-Plex PCR and API 20C AUX, MALDI-TOF MS, and rDNA Sequencing for a Wide Range of Clinically Isolated Yeast Species: Improved Identification by Combining 21-Plex PCR and API 20C AUX as an Alternative Strategy for Developing Countries

**DOI:** 10.3389/fcimb.2019.00176

**Published:** 2019-05-28

**Authors:** Amir Arastehfar, Farnaz Daneshnia, Mohammad Kord, Maryam Roudbary, Hossein Zarrinfar, Wenjie Fang, Sayed Jamal Hashemi, Mohammad Javad Najafzadeh, Sadegh Khodavaisy, Weihua Pan, Wanqing Liao, Hamid Badali, Sassan Rezaie, Kamiar Zomorodian, Ferry Hagen, Teun Boekhout

**Affiliations:** ^1^Yeast Biodiversity Department, Westerdijk Fungal Biodiversity Institute, Utrecht, Netherlands; ^2^Department of Medical Parasitology and Mycology, Tehran University of Medical Sciences, Tehran, Iran; ^3^Department of Medical Mycology and Parasitology, School of Medicine, Iran University of Medical Sciences, Tehran, Iran; ^4^Allergy Research Center, Mashhad University of Medical Sciences, Mashhad, Iran; ^5^Shanghai Key Laboratory of Molecular Medical Mycology, Department of Dermatology, Shanghai Institute of Medical Mycology, Shanghai Changzheng Hospital, Second Military Medical University, Shanghai, China; ^6^Food Microbiology Research Center, Tehran University of Medical Sciences, Tehran, Iran; ^7^Department of Parasitology and Mycology, School of Medicine, Mashhad University of Medical Sciences, Mashhad, Iran; ^8^Zoonoses Research Center, Kurdistan University of Medical Sciences, Sanandaj, Iran; ^9^Department of Medical Mycology, Invasive Fungi Research Center, School of Medicine, Mazandaran University of Medical Sciences, Sari, Iran; ^10^Department of Medical Mycology and Parasitology, Basic Sciences in Infectious Diseases Research Center, School of Medicine, Shiraz University of Medical Sciences, Shiraz, Iran; ^11^Yeast Biodiversity Department, Institute of Biodiversity and Ecosystem Dynamics, University of Amsterdam, Amsterdam, Netherlands

**Keywords:** API 20C AUX, 21-plex PCR, MALDI-TOF MS, LSU rDNA sequencing, developing countries

An author's name was incorrectly spelled as “Amir Aarstehfar.” The correct spelling is “Amir Arastehfar”.

The authors apologize for this error and state that this does not change the scientific conclusions of the article in any way. The original article has been updated.

